# Structural and Functional Abnormities of Amygdala and Prefrontal Cortex in Major Depressive Disorder With Suicide Attempts

**DOI:** 10.3389/fpsyt.2019.00923

**Published:** 2020-01-08

**Authors:** Lifei Wang, Yimeng Zhao, Elliot K. Edmiston, Fay Y. Womer, Ran Zhang, Pengfei Zhao, Xiaowei Jiang, Feng Wu, Lingtao Kong, Yifang Zhou, Yanqing Tang, Shengnan Wei

**Affiliations:** ^1^Department of Psychiatry, China Medical University, Shenyang, China; ^2^Department of Psychiatry, First Affiliated Hospital, China Medical University, Shenyang, China; ^3^Brain Function Research Section, First Affiliated Hospital, China Medical University, Shenyang, China; ^4^Department of Psychiatry, University of Pittsburgh School of Medicine, Pittsburgh, PA, United States; ^5^Department of Psychiatry, Washington University School of Medicine, St. Louis, MO, United States; ^6^Department of Radiology, First Affiliated Hospital, China Medical University, Shenyang, China; ^7^Department of Geriatric Medicine, First Affiliated Hospital, China Medical University, Shenyang, China

**Keywords:** major depressive disorder, suicide attempts, gray matter volume, functional connectivity, amygdala, prefrontal cortex

## Abstract

Finding neural features of suicide attempts (SA) in major depressive disorder (MDD) may be helpful in preventing suicidal behavior. The ventral and medial prefrontal cortex (PFC), as well as the amygdala form a circuit implicated in emotion regulation and the pathogenesis of MDD. The aim of this study was to identify whether patients with MDD who had a history of SA show structural and functional connectivity abnormalities in the amygdala and PFC relative to MDD patients without a history of SA. We measured gray matter volume in the amygdala and PFC and amygdala-PFC functional connectivity using structural and functional magnetic resonance imaging (MRI) in 158 participants [38 MDD patients with a history of SA, 60 MDD patients without a history of SA, and 60 healthy control (HC)]. MDD patients with a history of SA had decreased gray matter volume in the right and left amygdala (F = 30.270, P = 0.000), ventral/medial/dorsal PFC (F = 15.349, P = 0.000), and diminished functional connectivity between the bilateral amygdala and ventral and medial PFC regions (F = 22.467, P = 0.000), compared with individuals who had MDD without a history of SA, and the HC group. These findings provide evidence that the amygdala and PFC may be closely related to the pathogenesis of suicidal behavior in MDD and implicate the amygdala-ventral/medial PFC circuit as a potential target for suicide intervention.

## Introduction

Major depressive disorder (MDD) is one of the most common mental disorders (1), which is commonly associated with a higher suicide risk (2, 3). Previous studies have reported that patients with MDD had a 2%–12% lifetime risk of dying by suicide (4, 5), and the new study reported that the rated of suicide attempts (SA) was greater 4.78% in MDD by assessments of 3,284 adults with/without suicidal acts (6). Therefore, finding neurological features relating to SA in MDD patients could be a major achievement in preventing suicidal behavior (7).

The amygdala and prefrontal cortex (PFC) are two key brain regions which are responsible for processing emotional and cognitive information, especially emotional stimulation and executive function (8, 9). Recent structural and functional magnetic resonance imaging (MRI) studies demonstrate that the amygdala and PFC have been strongly implicated in MDD (10–17). For example, previous studies in MDD patients provided evidence of increased activation in the amygdala, and enlarged amygdala volume or reduced amygdala volume (10–14), which are mixed results due to different sample and method, so we will continue to explore the amygdala volume issue in this study. Regarding the PFC, morphological and functional alterations have been shown primarily in the orbitofrontal cortex, dorsolateral and ventrolateral PFC in MDD patients (12, 15–17). Depressed individuals also display decreased relationships between amygdala and dorsolateral PFC activity (12).

Neuroimaging studies suggest that abnormalities of the amygdala and PFC are closely related with SA in patients with MDD. For example, patients with a history of suicidal attempt have larger right amygdala volumes than nonsuicidal (18). Wagner and Blumberg and their colleagues found that MDD patients with prior SA showed significantly thinner cortex in the left dorsolateral, ventrolateral and prefrontal cortex in contrast to nonsuicide attempted patients (19, 20). MDD patients with a history of SA had lower orbitofrontal cortex gray matter volumes compared with those with no SA, or healthy comparison subjects (18, 21–23). A recent diffusion tensor imaging study showed that abnormal projections to the orbitofrontal cortex may disrupt affective and cognitive function, thereby conferring a heightened vulnerability for SA in MDD (24). However, few structural MRI studies have examined the gray matter volumes in amygdala and PFC in MDD with SA.

In addition to structural anomalies, there is also evidence for functional alterations in MDD patients with a history of suicidal behavior. Functional MRI studies found that MDD might be associated with a disturbed amygdala-PFC functional connectivity (FC). For example, MDD patients display decreased coupling between the amygdala and dorsolateral PFC (12, 25). Work from our lab has also shown altered amygdala-rostral, ventral, and dorsolateral PFC FC in MDD patients (26–28). Furthermore, patients who have attempted suicide show significant reductions in amygdala-prefrontal FC compared with a nonattempter patient group (29). With regard to MDD patients with SA, Kang and colleagues identified greater resting state FC in the amygdala in suicide attempters with MDD versus nonattempters, and there was greater connectivity of the left amygdala with the left superior OFC (30). So alterations in amygdala-PFC FC may be very important feature for MDD patients with SA. Understanding the PFC-amygdala neural circuitry with SA in MDD may be more beneficial to prevent suicide behavior.

The MRI literatures on suicide attempt in MDD suggested alterations of both structure and function in the amygdala-PFC neural circuitry. Compared with single neuroimaging analysis, the multiple neuroimaging analyses such as combining functional connectivity, regional gray matter volume, as well as combining amplitude of low-frequency fluctuations and white matter connectivity, could be used not only to explore the local functional and structural abnormalities, but also to identify more precisely the key neural circuitry in the mental disorders, providing the evidence to better understand the the mechanism of mental disorders in-depth (29, 31–33). Additionally, the latest studies suggested that data from multimodal fusion of structural and functional brain imaging analyses may be helpful to specifically predict symptoms and treatment effects in mental diseases (34, 35). Therefore, in the present study, we applied a multivariate model, which evaluate to more thoroughly characterize brain structural and functional abnormalities in MDD patient with SA. We hypothesized that patients with MDD who had a history of SA would demonstrate volumetric and FC differences in the amygdala and PFC relative to both HC and MDD patients without a history of SA, and we would perform to explore the relationships between the structural and functional findings across modalities.

## Materials and Methods

### Subjects

The study included 158 subjects aged 18–59 years: 38 MDD patients with a history of SA (mean age: 27.61 ± 10.536 years; 11 males), 60 MDD patients without a history of SA (mean age: 28.13 ± 7.617 years; 13 males), and 60 HC individuals (mean age: 25.83 ± 5.898 years; 17 males). Patients were divided into two groups: those with a history of SA [at least one attempt defined as a self-destructive act with some degree of intent to die (36)] and patients without a history of SA (nonattempters; no such history). Subjects were not considered suicide attempters if their self-injurious behavior was determined to have no suicidal intent. All patients were from Shenyang Mental Health Center and the Department of Psychiatry, First Affiliated Hospital of China Medical University, Shenyang, China. HC were recruited by advertising within the community. This study was approved by the Institutional Review Board of China Medical University. All participants provided written informed consent after receiving a detailed description of the study. All participants were evaluated by two trained psychiatrists to determine the presence or absence of Axis I psychiatric diagnoses using the Structured Clinical Interview for Diagnostic and Statistical Manual of Mental Disorders, Fourth Edition (DSM-IV) Axis I Disorders (SCID). MDD participants met DSM-IV diagnostic criteria for MDD and did not meet the criteria for any other Axis I disorder. HC participants did not have a current or lifetime Axis I disorder, or a history of psychosis, other Axis I disorders, or a history of SA in first-degree relatives as determined by a detailed family history. Participants were excluded if they had substance/alcohol abuse/dependence or a concomitant major medical disorder, any MRI contraindications, a history of head trauma with loss of consciousness for ≥5 min, or any neurological disorder. All subjects were evaluated using the Hamilton Depression Rating Scale (HAM-D) and Young Mania Rating Scale (YMRS).

### MRI Acquisition

MRI data were acquired using a GE signa HDX 3.0T scanner with a standard 8-channel head coil at the First Affiliated Hospital of China Medical University, Shenyang, China. Three-dimensional, high-resolution, T1-weighted images was collected using a 3D fast spoiled gradient-echo (FSPGR) sequence with the following parameters: TR/TE = 7.1/3.2 ms, image matrix = 240 × 240, field of view (FOV) = 240 mm^2^ × 240 mm^2^, 176 contiguous slices of 1mm without gap, voxel size = 1.0 mm^3^. We acquired fMRI images using a spin echo planar imaging (EPI) sequence, parallel to the anterior-posterior commissure plane with the following scan parameters: repetition time (TR) = 2000 ms; echo time (TE) > = 40 ms; image matrix = 64×64; field of view (FOV) = 24 cm^2^ × 24 cm^2^; 35 contiguous slices of 3 mm without gap; scan time = 6 min 40 s (the 6 min 40 s scans included a total of 200 volumes). We acquired a high-resolution structural image using a three-dimensional fast spoiled gradient-echo T1-weighted sequence: TR = 7.1 ms, TE = 3.2 ms, FOV = 24 cm × 24 cm, matrix = 240 × 240, slice thickness = 1.0 mm without gap, and 176 slices.

### Data Preprocessing

Structural brain images were processed using VBM8 toolbox (http://dbm.neuro.uni-jena.de/vbm8/), in Statistical Parametric Mapping 8 (SPM8; The Wellcome Department of Cognitive Neurology). VBM8 processing includes bias correction, tissue classification, and spatial normalization with Diffeomorphic Anatomical Registration Through Exponentiated Lie algebra (DARTEL) (37). Using the default parameters of VBM8, images were spatially normalized to the Montreal Neurological Institute (MNI) space to 1.5-mm^3^ voxel resolution. The modulation process was performed using nonlinear deformations employed for normalization that allowed for comparison of the absolute amount of tissue, corrected for individual brain sizes. Finally, all images were smoothed with an isotropic Gaussian kernel of 8-mm full width at half maximum (FWHM). These segmented, normalized, modulated, and spatially smoothed GM images were then used for subsequent VBM second-level statistical analysis.

Resting-state fMRI data preprocessing was carried out using Data Processing and Analysis for Brain Imaging software (DPABI; DPABI_V1.2_141101, http://rfmri.org/dpabi) (38), a toolbox for SPM8. The first ten volumes were discarded to allow for steady-state magnetization. Further data preprocessing included slice timing correction, head motion correction, spatial normalization, and smoothing. Spatial normalization was performed with the standard MNI EPI template. Spatial smoothing was completed with a 6-mm FWHM Gaussian filter. Data were then linearly detrended (39) and filtered using a band-pass temporal filter (0.01-0.08 Hz). We regressed for nuisance covariates, including the rigid-body 6 model, white matter signal, cerebrospinal fluid signal, and global signals. Subjects with head motion parameters exceeding 3 mm in displacement or 3° in rotation were excluded from the final analysis.

### Definition of Region of Interest

The Wake Forest University PickAtlas (http://fmri.wfubmc.edu/software/PickAtlas) was used to define the regions of interest for the PFC and amygdala. The PFC included the superior frontal gyrus, dorsolateral (Frontal_Sup), superior frontal gyrus, orbital part (Frontal_Sup_Orb), middle frontal gyrus (Frontal_Mid), middle frontal gyrus, orbital part (Frontal_Mid_Orb), inferior frontal gyrus, opercular part (Frontal_Inf_Oper), inferior frontal gyrus, triangular part (Frontal_Inf_Tri), inferior frontal gyrus, orbital part (Frontal_Inf_Orb), superior frontal gyrus, medial (Frontal_Sup_Medial), and superior frontal gyrus, medial orbital (Frontal_Mid_Orb), in the left and right hemispheres. We used the bilateral Automated Anatomical Labeling atlas template to define the amygdala regions of interest (40).

### FC Analysis

FC analysis was performed using correlation analysis between the seed amygdala ROI and PFC mask in a voxelwise manner using DPABI. The correlation coefficients were then transformed to z-values using the Fisher r-to-z transformation.

### Statistical Analysis

The demographic and clinical characteristics of the subjects were analyzed using IBM SPSS Statistics for Windows, Version 22.0 (Armonk, NY, USA). Student's t-tests, one-way analyses of variance, or Chi-square tests were used depending on the normality of the distribution and type of data. Categorical variables were described using frequencies and proportions. Statistical significance was determined by P < 0.05. Continuous variables were presented as mean ± standard deviation (P < 0.05, Bonferroni test). Partial correlation analyses (two-tailed), controlling for age and sex, were performed to explore the relationships between GMVs, FC, and symptom scores in the patient group (P < 0.05, Bonferroni correction). Additionally, we performed correlation analyses between GMVs/FC and HAM-D scores in MDD with SA group or in MDD without SA group in order to explore the association between GMVs/FC and the severity of attempt suicide, which could be evaluated by the HAM-D scores in MDD [(6, 41)] (P < 0.05, Bonferroni correction).

The GMVs of the bilateral PFC and amygdala were compared among the groups using one-way analysis of variance (ANOVA), with age and gender as covariates, using the general linear model in SPM8 (Wellcome Trust Centre for Neuroimaging, http://www.fil.ion.ucl.ac.uk/spm/software/spm8/). Amygdala-PFC FC was compared among the groups by one-way analysis of variance (One-way ANOVA), with age and gender as covariates, using Data Processing and Analysis for Brain Imaging software (DPABI; DPABI_V1.2_141101, http://rfmri.org/dpabi). Group differences were considered signiﬁcant for *p* values less than 0.001 (corrected, Gaussian random field [GRF] correction), and corresponding to a threshold of 69, 1, and 27 contiguous voxels for the GMVs of the bilateral PFC and amygdala and Z values of FC, respectively. We then extracted GMVs and Z values of FC for each cluster with significant differences for the three group comparison and conducted pairwise two sample t-tests, corrected for multiple comparisons (P < 0.05, Bonferroni test).

## Results

### Demographics and Clinical Characteristics

**Table 1** shows detailed participant demographic and clinical data. There were no significant differences among the three groups in terms of age (F = 1.374, P = 0.256), or sex (χ^2^ = 0.928, P = 0.629). There was no significant difference in illness duration (T = 1.105, P = 0.275), or medication status (χ^2^ = 0.582, P = 0.445) between the two MDD groups. As expected, three groups showed significant differences in HAM-D scores and YMRS scores (HAM-D: F = 83.907, P = .000; YMRS: F = 5.449, P = .005). Post hoc analyses found the MDD with and without a history of SA groups showed significantly higher HAM-D scores and YMRS scores than the HC group, however, HAM-D and YMRS scores demonstrated no significant differences between the two MDD groups.

**Table 1:** Demographics and clinical characteristics of all participants.

Variables	MDD with a history of SA (n = 38)	MDD without a history of SA (n = 60)	HC (n = 60)	F/χ^2^	*P*
Age (Mean ± SD)	27.61 ± 10.536	28.13 ± 7.617	25.83 ± 5.898	1.374	0.256
Gender, male (n, %)	11(28.95%)	13(31.67%)	17(28.33%)	0.928	0.629
Duration (months; Mean ± SD)	32.63 ± 83.17	17.17 ± 29.08		1.105^*^	0.275
Medication, yes (n, %)	22(57.90%)	30(50.00%)		0.582	0.445
HAM-D (Mean ± SD)	(n = 38)	(n = 58)	(n = 51)		
	18.55 ± 10.747	19.48 ± 9.289	1.02 ± 1.913	83.907	.000
YMRS (Mean ± SD)	(n = 37)	(n = 55)	(n = 50)		
	1.89 ± 4.074	1.87 ± 3.849	0.04 ± 0.198	5.449	0.005

*T values; MDD, major depressive disorder; HC, healthy controls; SA, suicide attempts; SD, standard deviation; HAM-D, Hamilton Depression Rating Scale; YMRS, Young Manic Rating Scale.

### Group Differences in GMV

**Figure 1:**
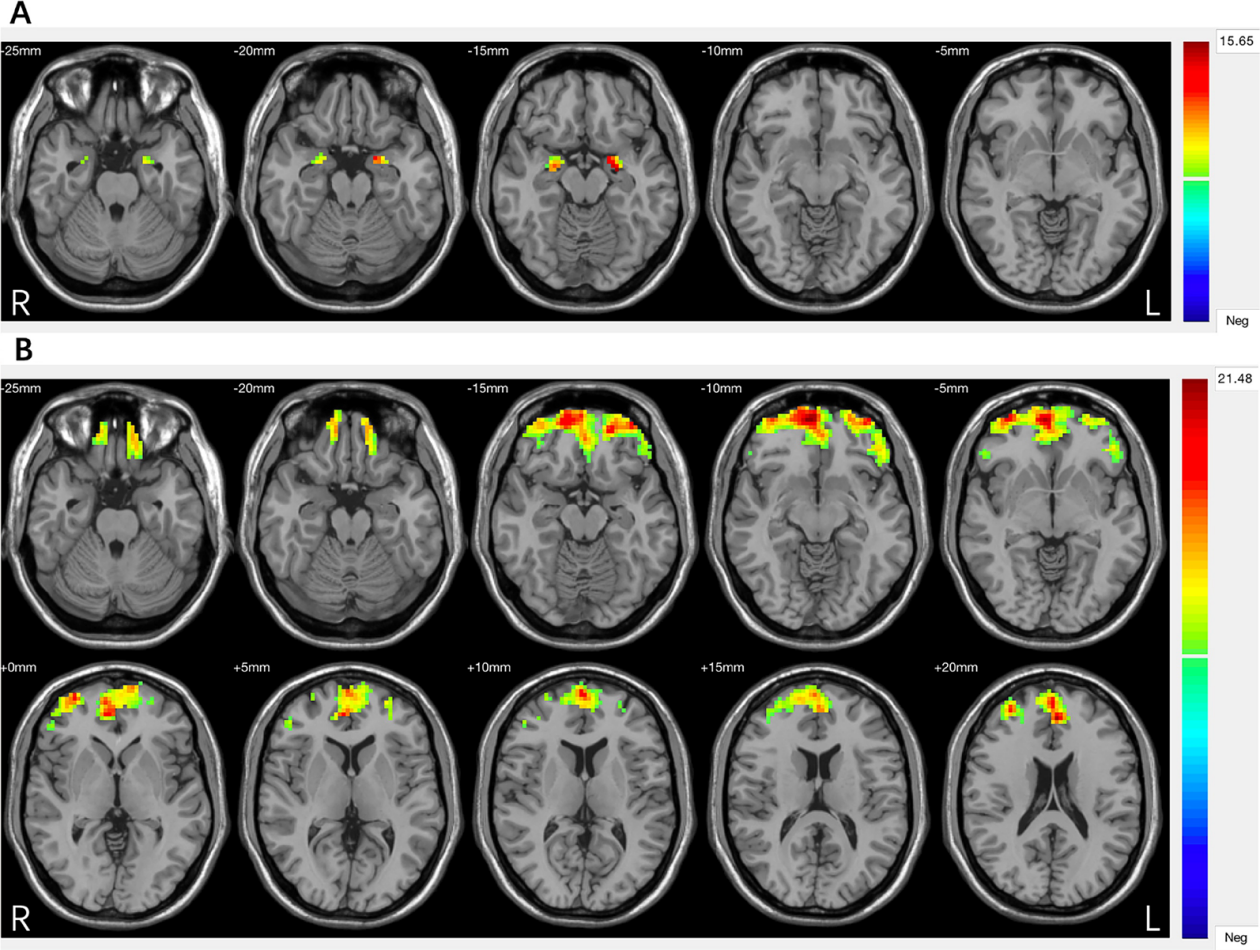
**(A)** Significant differences in grey matter volumes of the amygdala among the major depressive disorder (MDD) with a history of suicide attempts (SA), MDD without a history of SA, and healthy controls (HC) groups. **(B)** Significant differences in grey matter volumes of the prefrontal cortex (PFC) among the MDD with a history of SA, MDD without a history of SA, and HC groups. Significant at *P* < 0.001, corrected by Gaussian random field (GRF) correction.

**Figure 2:**
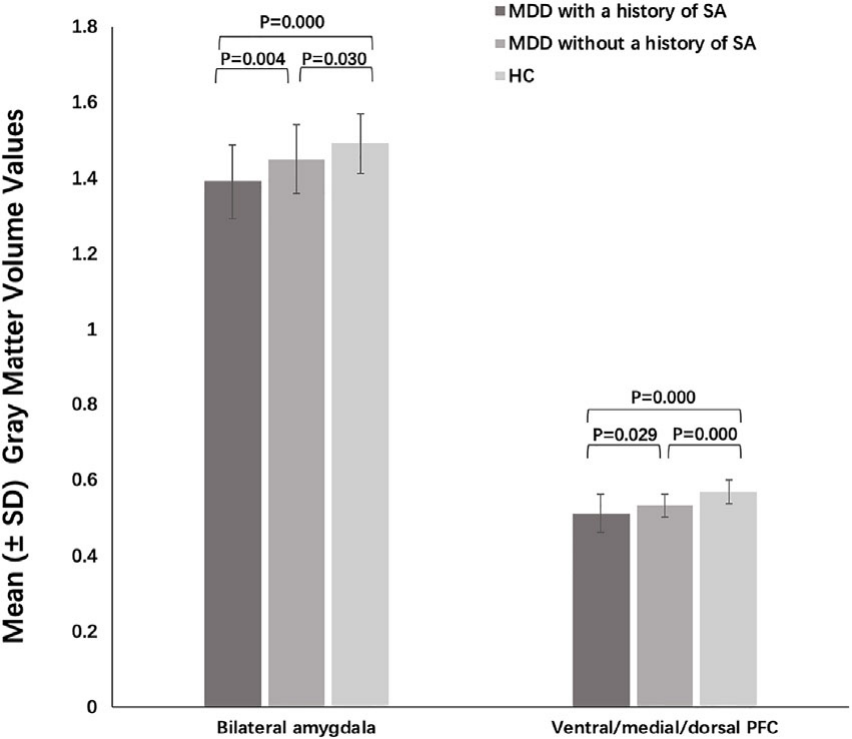
Post-hoc analysis of grey matter volumes of amygdala and prefrontal cortex (PFC) among the major depressive disorder (MDD) with a history of suicide attempts (SA), MDD without a history of SA, and HC groups.

Significant group differences were found in grey matter volumes in the bilateral amygdala (F = 30.270, P = 0.000) and in PFC regions including the ventral, medial, and dorsal PFC (F = 15.349, P = 0.000) (**Figure 1**, **Table 2**). Post hoc comparisons showed significant differences in GMV across all 3 groups (P < 0.03): HC > MDD without history of SA > MDD with history of SA for bilateral amygdala and ventral/medial/dorsal PFC volumes (**Figure 2**). Findings were unchanged with intracranial volume (ICV) as a covariate.

**Table 2:** Brain regions showing significant differences in amygdala and prefrontal cortex (PFC) between major depressive disorder (MDD) with and without a history of suicide attempts (SA) and healthy controls (HC) groups.

Index	Region	Voxel	MNI coordinates			F Values^*^
			X	Y	Z	
Amygdala	right amygdala	42	21	−6	−12	12.33
	left amygdala	44	−18	0	−18	15.30
PFC	left and right ventral PFC	2011	6	60	−9	21.14
	left and right medial PFC					
	left and right dorsal PFC					

MNI, Montreal Neurological Institute. *Significant at P < 0.001 corrected by Gaussian random field correction.

### Group Differences in FC

**Figure 3:**
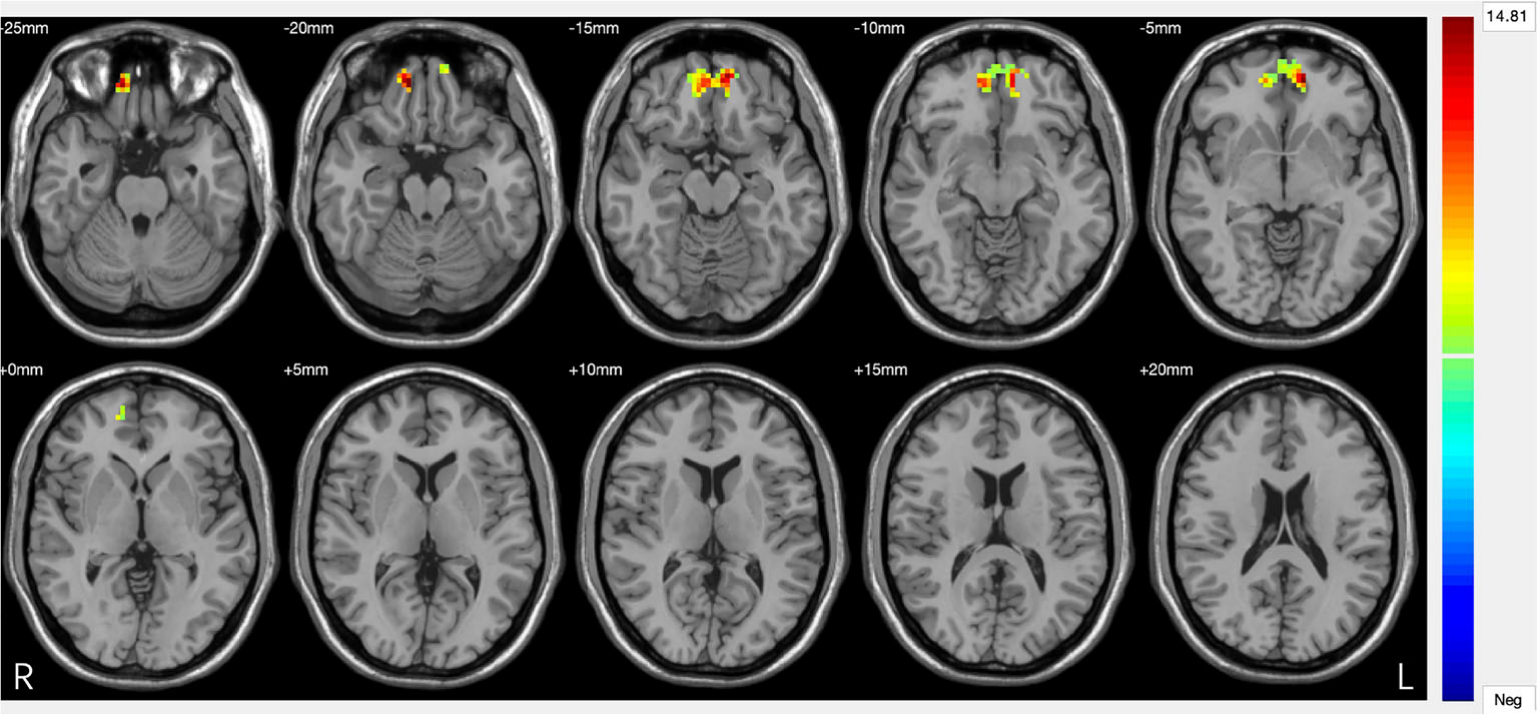
Significant differences in amygdala–prefrontal cortex (PFC) functional connectivity (FC) among the major depressive disorder (MDD) with a history of suicide attempts (SA), MDD without a history of SA, and HC groups. Significant at *P* < 0.001, corrected by Gaussian random field (GRF) correction.

**Figure 4:**
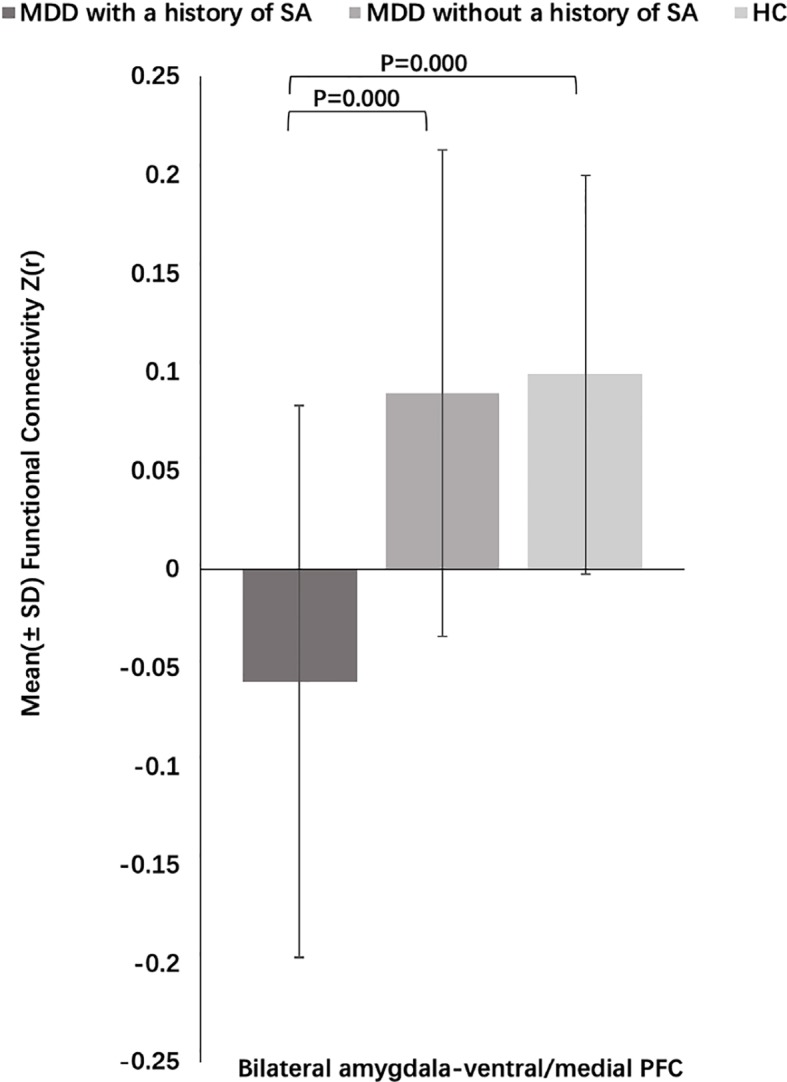
Post-hoc analysis of amygdala–prefrontal cortex (PFC) functional connectivity (FC) among the major depressive disorder (MDD) with a history of suicide attempts (SA), MDD without a history of SA, and HC groups.

Three-group analysis of FC showed significant differences in bilateral amygdala-left and right ventral PFC and bilateral amygdala-left and right medial PFC (F = 22.467, P = 0.000) (**Table 3**, **Figure 3**). MDD with a history of SA had significantly decreased FC in bilateral amygdala-left and right ventral PFC and bilateral amygdala-left and right medial PFC, compared with the HC (P = 0.000), and the MDD without a history of SA (P = 0.000), but no difference was observed in FC between the MDD without a history of SA and the HC group (P = 1.000) (**Figure 4**). Findings were unchanged with ICV as a covariate.

**Table 3:** Prefrontal cortex (PFC) regions showing significant changes from bilateral amygdala to PFC functional connectivity (FC) between major depressive disorder (MDD) with and without a history of suicide attempts (SA) and healthy controls (HC) groups.

Region	Voxel	MNI coordinates			F Values^*^
		X	Y	Z	
left and right ventral PFC	193	12	48	−21	14.81
left and right medial PFC					

MNI, Montreal Neurological Institute. *Significant at P < 0.001 corrected by Gaussian random field correction.

### Correlations Between GMV, FC, and Symptom Scores

No signiﬁcant correlations between the GMVs, FC, and symptom measures were observed in the MDD patient group (P > 0.05, Bonferroni correction). There were also no significant correlations between GMVs/FC and HAM-D scores in MDD with SA group or MDD without SA group, separately (P > 0.05, Bonferroni correction). We used the left and right amygdala separately as the seed ROI, the GMVs of left and right amygdala and the left and right amygdala-PFC functional connectivity were compared among the groups. Additionally, the subjects were divided into males and females, and the structural and functional changes were compared among three groups separately. We have added these detailed analyses (**Supplementary Material**).

## Discussion

We report that MDD patients with a history of SA have decreased gray matter volume in the amygdala and PFC, and reduced functional connectivity from the amygdala to PFC, compared to both HC and individuals with MDD without a history of SA. Structural differences occurred in a graded fashion, such that MDD with a history of SA showed the lowest volumes, then MDD without a history of SA, then the HC group. In contrast, functional connectivity differences were only present between the MDD SA group and the MDD without SA group; there were no significant differences between the MDD without SA and the HC groups. We discuss the implications of these findings below.

The amygdala has a well-documented role in emotion processing (8, 42). We found reduced amygdala volume in patients with a history of SA, consistent with 43). A recent meta-analysis found decreased GMV in subcortical structures in MDD with suicidal ideation and attempt compared with controls (44), also in line with our findings of decreased amygdala and PFC GM volumes. However, larger amygdala volumes have been shown in MDD females with suicidal ideation compared with nonsuicidal MDD (18). The inconsistency may relate to differences in patient characteristics (both males and females with MDD were included in this study). Future studies specifically examining sex differences in the amygdala in MDD and suicidal behavior are warranted. Several prior studies have found physiological differences in the amygdala in MDD patients who have attempted or died by suicide compared with individuals who have not, implicating the amygdala as a potential neurobiological substrate for suicidal behavior in MDD. Ballard et al. suggest that the amygdala may mediate fear-potentiated startle in MDD individuals with lifetime history of suicide attempt (45). In addition, reductions in the messenger RNA levels of multiple transcripts of QKI in the amygdala have been found in suicide victims compared with control subject (46), and significantly lower numbers of a 2A-adrenoceptors in the amygdala of depressed suicide completers compared with controls (47).

The PFC may be an important associated with suicidal behavior, particularly in the ventral PFC, medial PFC, and dorsal PFC (18, 48–51). The current evidence in adults who have attempted suicide supports ventral prefrontal volume decreases and dysfunction have been linked to lethality (52). Prefrontal localized hypofunction and impaired serotonergic responsivity are proportional to the lethality of the suicide attempt in depressed patients, especially in the ventral, medial, and lateral PFC (53). Suicide action was associated with abnormal activity in the medial PFC (54). In our study, significant volume decreases in the ventral, medial, and dorsal PFC regions were found in suicide attempters with MDD.

Prior studies have reduced GMV in the ventral PFC and medial PFC in MDD individuals with prior suicide attempters (18, 29, 43, 49, 50, 55, 56). Other studies have found reduced GVM in orbitofrontal cortex in MDD patients with a history of SA, compared with those without SA and HC subjects (18, 21–23). Thus, alterations in PFC GMV are implicated in suicidal behavior in MDD patients.

FC between the bilateral amygdala and ventral and medial PFC regions was decreased in MDD patients with a history of SA, compared to MDD patients with a history of SA and HC groups in this study. Our findings are supported by prior finding of decreased FC between the amygdala and ventral PFC in suicide attempters (29). However, increased FC between the left amygdala with left superior OFC has been observed in suicide attempters with MDD versus nonattempter (30). Differences in sample size (this study had larger sample size) and methodology (different amygdala seed regions) may contribute to differences in findings. Nevertheless, these findings altogether suggest that abnormalities in amygdala-ventral/medial PFC neural circuitry may relate to suicidal behavior and could provide insight into the mechanisms underlying suicidality in MDD. In this study, amygdala-ventral/medial PFC FC was lower in the MDD without a history of SA but not statistically different when compared to the HC group. Prior studies, including work from this group, has found alterations in FC between the amygdala and ventral/medial PFC in MDD (27, 28, 57). So whether the functional neural basis from the amygdala to the PFC is related to the pathophysiology of MDD need the further study.

There are several limitations to this study. Firstly, the sample size was not large enough, but we are continuing to collect relevant samples for future research. Secondly, some patients are prescribed psychotropic medications or demonstrate a longer duration of illness, which may affect the accuracy of the results. Thirdly, our study excluded the patients with other psychiatric comorbidities. While this provided sample homogeneity and likely improved our ability to detect significant effects, the generalizability of our findings is unclear as the majority MDD patients have other psychiatric comorbidities. Fourthly, we did not collect other information to assess severity of suicide ideation and suicide attempt (e.g., Suicide Intent Scale (SIS), and Columbia Suicide Severity Rating Scale (C-SSRS)), so we did not know what our findings are related to the severity of SA. Fourthly, in our study we did not collect information on the numbers of SA associated with the severity attempts. It is an important to record the times of SA and assess the severity of depression related to severity attempts to better understand the severity of SA. Finally, our study had no specific assessment of the impulse scale associated with suicidal behavior in our study. Further studies addressing these limitations are needed for more definitive understanding of complex relationship between neural structure and function and suicidal behavior in MDD.

## Conclusions

MDD patients with a history of SA exhibited decreased GMV in the amygdala, and ventral/medial/dorsal PFC, as well as reduced FC between amygdala and ventral/medial PFC. These findings indicate structural and functional alterations in the amygdala in suicidal behavior in MDD. Moreover, the study highlights the importance of the amygdala and PFC circuitry in suicidal behavior in MDD and implicates the amygdala-ventral/medial PFC circuit as a potential target for suicide intervention.

## Data Availability Statement

The datasets for this study are available on request to the corresponding authors.

## Ethics Statement

This study was approved by the Institutional Review Board of the China Medical University and was performed according to the principles of the Declaration of Helsinki. All participants provided written informed consent after receiving a detailed description of the study.

## Author Contributions

SW and YT designed the experiment. RZ, PZ, XJ, LK, FW, and YMZ carried it out. LW, SW, and YMZ analyzed the data. LW, YFZ, and SW wrote the manuscript. EE and FYW edited the manuscript. All the authors discussed the results and reviewed the manuscript.

## Funding

This work was supported by grants from the National Natural Science Foundation of China (81701336 to SW; 81271499 and 81571311 to YT), the Liaoning Education Foundation (L2015591 to SW), and National Key Research and Development Program (2016YFC1306900 to YT). We would like to thank the patients and family members who contributed so much to this study and the First Affiliated Hospital of China Medical University for its active support of the project.

## Conflict of Interest

The authors declare that the research was conducted in the absence of any commercial or financial relationships that could be construed as a potential conflict of interest.
