# Supplementary material for: Structural and Functional Abnormities of Amygdala and Prefrontal Cortex in Major Depressive Disorder With Suicide Attempts
Source: Front Psychiatry. 2020 Jan 8;10:923. doi: 10.3389/fpsyt.2019.00923 (PMC6960126; doi:10.3389/fpsyt.2019.00923)
Supplement: Supplementary file 1 [file DataSheet_1.pdf]

Supplemental Material

Section part1

Methods

We used the left and right amygdala separately as the seed ROI, The GMVs of left and right amygdala were compared among the groups using one-way analysis of variance (ANOVA), with age and gender as covariates, using the general linear model in SPM8 (Wellcome Trust Centre for Neuroimaging, http://www.fil.ion.ucl.ac.uk/spm/software/spm8/). Then, we used the difference PFC regions as a mask found in FC between bilateral amygdala left and right ventral/medial PFC in our original text. The left and right amygdala-PFC functional connectivity was compared among the groups by one-way analysis of variance (One-way ANOVA), with age and gender as covariates, using Data Processing and Analysis for Brain Imaging software (DPABI; DPABI_V1.2_141101, http://rfmri.org/dpabi). Group differences were considered significant for p values less than 0.001 (corrected, Gaussian random field [GRF] correction). We then extracted GMVs and Z values of FC for each cluster with significant differences for the three group comparison and conducted pairwise two sample t-tests, corrected for multiple comparisons ($p < 0.05$, Bonferroni test).

Results

The trends of the average of left/right amygdala gray matter volume among three groups were consistent with the trend of bilateral amygdala in our original text. A post hoc analysis of GM values showed significant differences in all regions among the three groups (Figure S1). The findings of the significant difference between groups was consistent with those in our original text, only there was no significant difference between MDD without SA and HC group in left amygdala gray matter volume (P=0.151). The trends of the average of FC values between left/right amygdala and left and right ventral/medial PFC among three groups were consistent with the trend of between bilateral amygdala and left and right ventral/medial PFC in our original text. A post hoc analysis of Z values of FC showed significant differences in all regions among the three groups (Figure S2). The results of the significant difference between groups in FC between right amygdala and left and right ventral/medial PFC was consistent with those in our original text, however, there was no significant difference

among three groups in FC between left amygdala and left and right ventral/medial PFC (P=0.055).

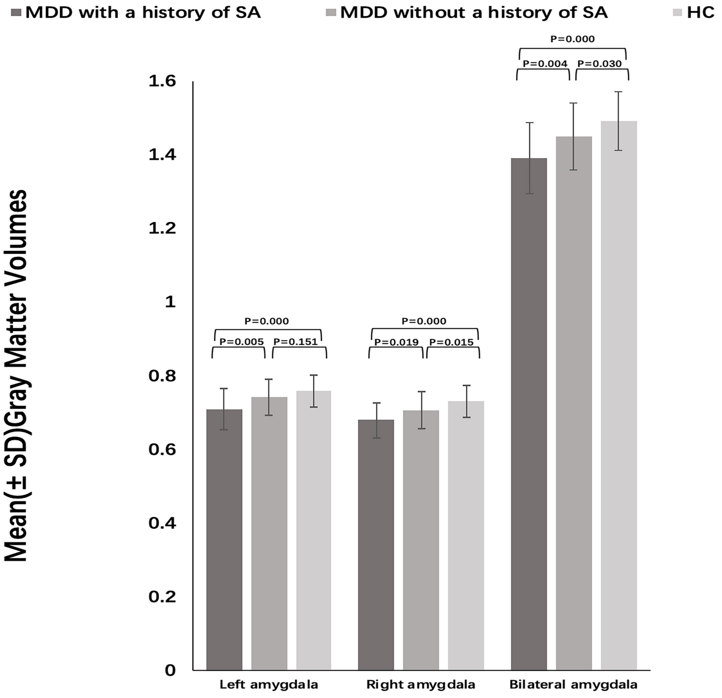

Figure S1. Post-hoc analysis of grey matter volumes of left/right and bilateral amygdala among the major depressive disorder (MDD) with a history of suicide attempts (SA), MDD without a history of SA, and HC groups.

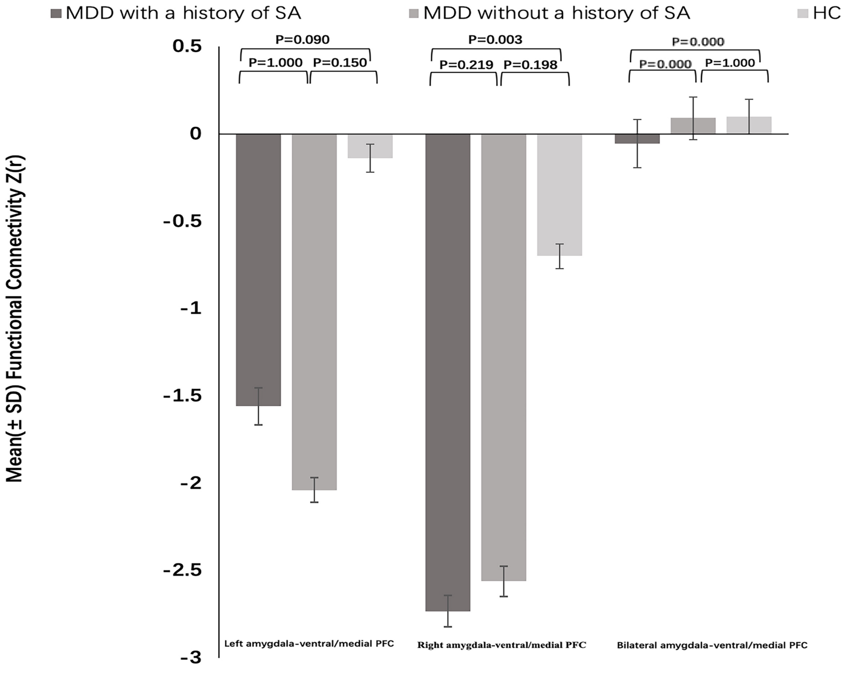

Figure S2. Post-hoc analysis of left/right-PFC functional connectivity (FC) and bilateral amygdala-PFC FC among

the major depressive disorder (MDD) with a history of suicide attempts (SA), MDD without a history of SA, and HC groups.

Section part2

According to the reviewer's suggestion, on the one hand, the subjects were divided into males and females, and the structural and functional changes were compared among three groups (male MDD with SA, male MDD without SA, and male HC), and among another three groups (female MDD with SA, female MDD without SA, and female HC), separately. On the other hand, for age, partial correlation analyses (two-tailed) were performed to explore the relationships between the age, GMVs and FC in each group, separately. No significant correlations between the age, GMVs and FC were observed (P>0.05, partial correlation analysis). These results are consistent with our findings in our original text. The detailed results were as follows.

Firstly, if sex=male, 41 male participants were divided into 11 male MDD patients with SA group, 13 male MDD patients without SA group, and 17 male HC group. Significant three-group differences were found in grey matter volumes (GMV) in the bilateral amygdala (F=8.080, P=0.001; GMVs(mean±sd): male MDD with SA(1.347±0.113), male MDD without SA(1.446±0.093), male HC(1.505±0.100)), in PFC regions including the ventral, medial, and dorsal PFC (F=11.084, P=0.000; GMVs(mean±sd): male MDD with SA(0.502±0.060), male MDD without SA(0.520±0.026), male HC(0.569±0.032)), and FC in bilateral amygdala-left and right ventral PFC and bilateral amygdala-left and right medial PFC (F=6.097, P=0.005; Z values of FC (mean±sd): male MDD with SA(-0.042±0.139), male MDD without SA(1.131±0.128), male HC(0.086±0.113)). Post hoc comparisons showed significant differences in GMV and FC across all 3 groups: male MDD with SA < male HC for bilateral amygdala (P=0.001) and ventral/medial/dorsal PFC volumes(P=0.000); male MDD without SA < male HC for ventral/medial/dorsal PFC volumes (P=0.005); male MDD with SA < male MDD without SA (P=0.005) or male MDD with SA < male HC (P=0.035) for FC in bilateral amygdala-left and right ventral PFC and bilateral amygdala-left and right medial PFC. Secondly, if sex=female, 117 female participants were divided into 27 female MDD patients with SA group, 47 female MDD patients without SA group, and 43 female HC group. Significant three-group differences were

found in grey matter volumes (GMV) in the bilateral amygdala (F=7.509, P=0.001; GMVs(mean±sd): female MDD with SA(1.408±0.086), female MDD without SA(1.451±0.092), female HC(1.487±0.070)), in PFC regions including the ventral, medial, and dorsal PFC (F=19.421, P=0.000; GMVs(mean±sd): female MDD with SA(0.518±0.046), female MDD without SA(0.537±0.030), female HC(0.570±0.033)), and FC in bilateral amygdala-left and right ventral PFC and bilateral amygdala-left and right medial PFC (F=17.399, P=0.000; Z values of FC (mean±sd): female MDD with SA(-0.063±0.145), female MDD without SA(0.077±0.122), female HC(0.103±0.098)). Post hoc comparisons showed significant differences in GMV and FC across all 3 groups: female MDD with SA < female HC for bilateral amygdala (P=0.001) and ventral/medial/dorsal PFC volumes(P=0.000); female MDD without SA < female HC for ventral/medial/dorsal PFC volumes (P=0.000); female MDD with SA <female MDD without SA (P=0.000) or female MDD with SA < female HC (P=0.000) for FC in bilateral amygdala-left and right ventral PFC and bilateral amygdala-left and right medial PFC.

For age, partial correlation analyses (two-tailed), controlling for sex, were performed to explore the relationships between age, GMVs and FC in each group, separately. The results showed that no significant correlations between age and GMVs in bilateral amygdala (MDD with SA group: r=-0.211, P=0.211; MDD without SA group: r=0.063, P=0.633; HC group: r=0.056, P=0.672), in ventral/medial/dorsal PFC volumes (MDD with SA group: r=-0.063, P=0.709; MDD without SA group: r=-0.020, P=0.880; HC group: r=0.150, P=0.258), and in FC in bilateral amygdala-left and right ventral PFC and bilateral amygdala-left and right medial PFC(MDD with SA group: r=-0.254, P=0.130; MDD without SA group: r=-0.021, P=0.876; HC group: r=-0.007, P=0.956).
